# Fluensulfone and 1,3-dichloroprene for plant-parasitic nematode management in potato production

**DOI:** 10.21307/jofnem-2019-038

**Published:** 2019-07-23

**Authors:** Zane J. Grabau, Joseph W. Noling, Pablo A. Navia Gine

**Affiliations:** 1 Entomology and Nematology Department, University of Florida, 1881 Natural Area Drive, Gainesville, FL, 32601; 2 Citrus Research and Education Center, University of Florida; 3 ADAMA Agricultural Solutions Ltd., 3120 Highwoods Blvd. #100 Raleigh, NC, 27604

**Keywords:** Potato, *Solanum tuberosum*, Sting nematode, *Belonolaimus longicaudatus*, Lesion nematode, *Pratylenchus*, Stubby-root nematode, Fluensulfone, 1,3-dichloropropene, Nematicide, Nematode management

## Abstract

Florida produces 35% of the spring potato (*Solanum tuberosum*) crop in the USA, but plant-parasitic nematodes suppress yield in the region. The stubby-root nematodes, *Paratrichodorus* (*Nanidorus*) spp. and *Trichodorus* spp., vectors for corky ringspot disease, and sting nematode (*Belonolaimus longicaudatus*) are among the most damaging nematodes in Florida potato production. Nematicide application is an important component of nematode management in this system, but relatively few nematicides are currently available. Therefore, pre-plant applications of fluensulfone nematicide at various rates (3, 4, 6, and 8 l/ha) and the commercial standard fumigant 1,3-dichloropropene (1,3-D) were tested for management of plant-parasitic nematodes in three field trials from 2016 to 2018. Both fluensulfone, at all rates, and 1,3-D consistently decreased sting nematode abundance relative to the untreated control at harvest. Neither fluensulfone nor 1,3-D affected stubby-root nematode abundances at harvest. Efficacy of fluensulfone and 1,3-D for lesion nematode (*Pratylenchus* sp.) management varied by year. In 2016 and 2018, fluensulfone at most rates and 1,3-D increased marketable potato yield relative to the untreated control with increases by 49 to 66% and 33 to 55% in 2016 and 2018, respectively. In 2017, fluensulfone at lower rates (3, 4, and 6 l/ha) increased marketable potato yield relative to the untreated control by 41 to 61%, but fluensulfone at 8 l/ha and 1,3-D had similar yields to the untreated control. Results suggest that nematicidal activity of fluensulfone and 1,3-D varies by target nematode with both products effective against sting nematode, ineffective against stubby-root nematodes, and inconsistent against lesion nematode. In conclusion, fluensulfone and 1,3-D are effective options for sting nematode management in Florida potato production.

Potato (*Solanum tuberosum*) is a staple crop and food source in many places throughout the world. In the USA, Florida is a key provider of potatoes during the spring season. In 2016, Florida produced 274 million kg of potatoes, worth $48 million, which constituted 35% of the spring crop in the USA by volume (NASS-USDA, 2017). Potatoes are grown in many parts of the Florida peninsula, but approximately 66% of acreage is in three counties in Northeastern Florida (NASS-USDA, 2014). Plant-parasitic nematodes are a major problem in this warm area with coastal sandy soils (Weingartner et al., 1993; Crow et al., 2000a). A wide variety of plant-parasitic nematodes are abundant in the area, but sting nematode (*Belonolaimus longicaudatus*) and the stubby-root nematodes, *Paratrichodorus* (*Nanidorus*) spp. and *Trichodorus* spp., are the most problematic (Perez et al., 2000; Crow et al., 2000b). Sting nematode is a very damaging pathogen of potato that stunts the root system, reducing tuber yield (Weingartner et al., 1993). The economic damage threshold for sting nematode in Florida potato production, based on a two-year research trial, is at or near the detection limit in pre-plant soil (Crow et al., 2000b). Based on the same study, each sting nematode detected per 130 cm^−3^ soil suppresses potato yield by 199 kg/ha.

Stubby-root nematodes are not thought to cause much direct damage to potato (Weingartner et al., 1993; Crow et al., 2000a), but they vector tobacco rattle virus (Perez et al., 2000; Brown et al., 2009), the causal agent of corky ringspot disease (CRS). Corky ringspot disease has been confirmed in Northeast Florida and is a recurring problem with yield suppression of 25% reported in some trials (Weingartner and Shumaker, 1990c; Perez et al., 2000). Potatoes infected with CRS have mottled skin and internal arcs or rings of necrosis making them unmarketable (Weingartner et al., 1993). Symptoms of CRS are correlated with stubby-root nematode abundance, particularly early in the growing season, so management of this nematode is an important strategy for CRS management (Perez et al., 2000).

There are relatively few nematode management options for potato producers in Northeast Florida. Sting and stubby-root nematodes have wide host ranges making it difficult to manage these nematodes with crop rotation or cover cropping (Crow et al., 2000a, 2001). Production constraints for Northeast Florida potato producers – such as specialized equipment, the need for high-value rotation crops, and a potato growing season that extends into early summer – also limit the number of viable rotation cash crops or cover crops. Additionally, crop rotation apparently does not eliminate CRS as the disease can persist in fields that have been rotated out of potato for many years (Weingartner and Shumaker, 1990c). There are some CRS resistant potato cultivars available that exhibit reduced incidence of CRS (Weingartner et al., 1993; Brown et al., 2009), but sting nematode resistant or tolerant cultivars are not available. Because of the limitations of other management strategies and the high pressure from nematodes, fumigant and non-fumigant nematicide application is an important component of nematode management in Florida potato production.

The limited number of nematicides labeled and available for Florida potato production is also a challenge for nematode management. In recent years, growers have temporarily or permanently lost use of some nematicides, such as oxamyl and aldicarb, due to registration cancellations or production interruption. Oxamyl and aldicarb have been important products for control of stubby-root nematodes and CRS as they have provided good control of these pathogens; often better control than fumigants (Weingartner and Shumaker, 1990a, 1990b; Weingartner et al., 1993). This has left growers reliant on a limited number of nematicides, particularly the fumigant 1,3-dichloropropene (1,3-D), or a combination of 1,3-D and ethoprop when CRS is present. Therefore, it is important to identify viable alternative nematicides for nematode management in potato production. Fluensulfone (NIMITZ^®^, ADAMA) is a relatively new nematicide in the fluoroalkenyl group. It has irreversible nematicide activity with a different mode of action than organophosphate or carbamate non-fumigant nematicides (Oka et al., 2012; Kearn et al., 2014, 2017). Fluensulfone has been effective for managing root-knot nematodes (*Meloidogyne* spp.) in various vegetable crops in lab (Oka et al., 2013), greenhouse (Jones et al., 2017), and field studies (Morris et al., 2015, 2016). It was also relatively effective at managing *Globodera pallida* (potato cyst nematode) in field trials (Norshie et al., 2016). There is little published research on the efficacy of fluensulfone against other nematodes and no previous reports on fluensulfone efficacy against sting or stubby-root nematodes in potato production to our knowledge.

Based on these needs, the objectives of this study were to evaluate the efficacy of fluensulfone at various rates and the fumigant 1,3-D for (i) management of plant-parasitic nematodes and (ii) potato yield response in Florida potato production.

## Materials and methods

### Field site and experimental design

The field trials were located at the University of Florida Hastings Agricultural Education Center in Hastings, Florida (29.692, −81.441). Soil at the field site was an Ellzey fine sand (sandy, silicaceous, hyperthermic Arenic Ochraqualf) with 95% sand, 2% silt, 3% clay, and <1% organic matter. The experimental units were field plots of four rows spaced 102 cm apart and 26 m long. The study was a randomized complete block design with six replicates and a single factor – nematicide application. The study was conducted in 2016, 2017, and 2018 at the same field site and treatments were not re-randomized each year. There were six nematicide application treatments as describe in Table 1: (i) fluensulfone at 1.40 kg a.i./ha, (ii) fluensulfone at 1.96 kg a.i./ha, (iii) fluensulfone at 2.80 kg a.i./ha, (iv) fluensulfone at 3.92 kg/ha, (v) 1,3-D at 60.90 l/ha, and (vi) untreated control.

**Table 1.** Nematicide application treatment rates and application methods.

Treatment number	Product	Active ingredient	Total product rate (l/ha)	Active ingredient rate (kg a.i./ha)	Application method
1	Nimitz	Fluensulfone	2.92	1.40	Boom-applied, chopper-incorporated to 15 cm
2	Nimitz	Fluensulfone	4.11	1.96	Boom-applied, chopper-incorporated to 15 cm
3	Nimitz	Fluensulfone	5.87	2.80	Boom-applied, chopper-incorporated to 15 cm
4	Nimitz	Fluensulfone	8.20	3.92	Boom-applied, chopper-incorporated to 15 cm
5	Telone II	1,3-dichloropropene	60.90	–	Injected 25 cm deep, 1 shank per bed (102 cm wide)
6	Untreated control				

Nematicide applications were made in early January, two to four weeks before planting. In 2016, 2017, and 2018, nematicides were applied 21, 15, and 27 d before planting, respectively. The time between nematicide application and planting varied by year based on weather conditions, which limited the ability to access the field for planting. See Table 2 for specific dates of nematicide application and other trial maintenance tasks. Before nematicide application, the field was chopped using a rotary-type spade to a depth of 15 cm. Fluensulfone treatments were applied on a broadcast basis across all four rows of the selected plots using tractor-driven spray boom delivered via flat fan nozzles. Nozzles were located 51 cm or less above ground, according to label instructions. Following fluensulfone application, the field was tilled to a depth of 15 cm again using a rotary spade, immediately followed by constructing hilled beds using two bedding discs per bed, and shaping beds with a bed shaper to press and flatten beds.

**Table 2.** Schedule for data collection and trial maintenance from 2016 to 2018.

Task	2016	2017	2018
Pre-plant soil samples	January 25	January 3	January 10
Nematicides applied	January 25	January 3 to 4	January 10
Potatoes planted	February 15	January 19	February 6
Harvest soil samples	May 31	May 2	May 7
Potatoes harvested	May 31	May 2	May 23 to 24

In plots that received 1,3-D treatment, immediately following pre-plant tillage, 1,3-D fumigant was injected into the soil to 25 cm via shanks spaced at 102-cm intervals to achieve in-row treatments. Immediately following fumigant injection, the beds were formed and shaped. Rainfall, soil, and air temperature for the four weeks before and after nematicide application in 2016 to 2018 is provided in Table 3 as moisture and temperature may affect pesticide efficacy. Rainfall and temperature information were collected from the Florida Automated Weather Network weather station at the Hastings Agricultural Extension Center where this study was conducted. Conditions were moist when nematicides were applied in both 2016 and 2018, but somewhat drier in 2017. In all three years, 2 cm or more rainfall occurred within 3 d after nematicide application – a factor that is important because irrigation or rainfall is recommended after fluensulfone application to help incorporate the product into soil.

**Table 3.** Rainfall, soil, and air temperature at trial site before and after nematicide application in 2016, 2017, and 2018.

	2016			2017			2018		
		Temperature (C)			Temperature (C)			Temperature (C)	
Time period^a^	Rainfall^b^	Soil^c^	Air^d^	Rainfall	Soil	Air	Rainfall	Soil	Air
4 WBA	2.29	21.1	19.9	0.00	18.3	15.6	0.00	15.7	15.1
3 WBA	0.61	17.0	13.3	0.69	19.1	17.1	0.15	18.0	16.8
2 WBA	2.46	15.5	11.3	0.08	18.2	16.6	0.18	14.9	9.6
1 WBA	2.13	13.4	8.3	0.25	18.3	17.2	3.73	11.2	7.5
Day nematicide applied	0.00	12.0	8.9	0.00	19.6	19.0	1.65	15.9	18.5
1 WAA	2.67	15.0	14.6	2.16	16.3	11.6	1.45	14.5	11.6
2 WAA	7.24	15.9	14.6	0.08	17.8	18.2	0.53	13.1	11.6
3 WAA	0.00	13.2	11.6	0.08	18.6	18.4	8.94	15.0	14.3
4 WAA	2.51	15.9	15.5	0.00	16.8	12.4	0.30	15.0	15.1

Note: ^a^Weeks before nematicide application (WBA) or weeks after nematicide application (WAA); ^b^rainfall (cm) measured at 2 m; csoil temperature at 10 cm below soil surface; dair temperature at 60 cm

Potato seed pieces (Red LaSoda cultivar) were planted approximately three weeks later (Table 2). The trial was maintained uniformly with standard fertilizer and pest management practices for the region (Zotarelli et al., 2017). Plots were irrigated by subsurface tile drainage as needed throughout potato production. Sorghum-sudangrass (*Sorghum bicolor*) was grown as a summer cover crop before each season. This is the standard cover crop for the region and is susceptible to most of the nematodes present in this study, so growing it helped maintain nematode populations. Potatoes were harvested in late May or early June (Table 2) from 6.1 m of the center two rows of each plot using a single-row digger. All potatoes harvested from these sections were graded and weighed by size class for marketable tubers and damage category for cull, unmarketable tubers. The marketable size classes were A1 (8.4-10.2 cm diameter), A2 (6.4-8.3 cm), A3 (4.8-6.3 cm), B (3.8-4.7 cm), and C (1.3-3.7 cm). Total yield for all grade A potatoes was also calculated and abbreviated as A123. Any potatoes that were smaller than size class C, rotten, green from sunburn, had growth cracks, or were misshapen were considered culls and cull weights in each category were weighed. A 20-potatoes subsample from each plot was also assessed for internal defects including corky ringspot, internal heat necrosis, and hollow heart.

### Soil sampling and nematode quantification

Soil samples were collected for nematode analysis before nematicide application and around harvest (Table 2). In each plot, 12 soil cores to a depth of 30 cm and 2 cm diameter were collected using an Oakfield tube. Cores were collected from the center two hilled beds in each plot. At harvest, cores were collected close to potato plants and cores were collected from bare ground before planting since beds were fallow. Samples were homogenized in plastic bags and nematodes were extracted from 100 cm^3^ soil. In 2016 and 2017, nematodes were extracted by Baermann funnel (Southey, 1986) whereas in 2018, nematodes were extracted by sucrose floatation and centrifugation (Byrd et al., 1976). After nematodes were extracted, plant-parasitic nematodes were identified to genera and quantified using an inverted, light microscope. Sting nematode, lesion nematode (*Pratylenchus* sp.), and stubby-root nematode were present across the sites in each year of the study. Stunt nematodes (*Tylenchorhynchus* sp.) were present across the site in 2018.

### Statistical analysis

Response variables (nematode abundances and potato yield or culls by category) were analyzed by separately by year and season because nematode extraction methods differed by year and treatment effects varied by year and season. Response variables were subjected to ANOVA using SAS. Response variables were not transformed before analysis. For variables with significant (*P* < 0.05) treatment effects in ANOVA, means were separated by Fisher’s least significant differences at *α* = 0.05.

## Results

### Nematode abundances

**Figure 1:**
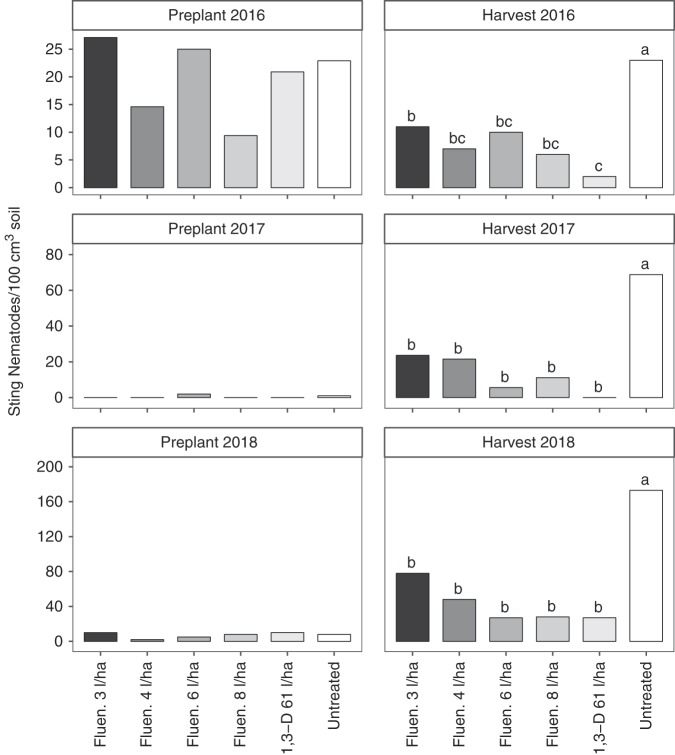
Sting nematode (*Belonolaimus longicaudatus*) abundance as affected by nematicide treatments. The abbreviations Fluen. and 1,3-D denote fluenosulfone (Nimitz EC) and 1,3-dichloropropene nematicide applications, respectively. Different letters indicate significantly different means based on Fisher’s protected LSD at *α* = 0.05. Bars represent means of six replicates.

Sting nematode abundance before planting was not significantly different among nematicide treatments in any year (Fig. 1). In all three years, sting nematode abundance at harvest were significantly greater in the untreated control than any of the 1,3-D or fluensulfone treatments. In 2017 and 2018, at harvest there were no significant differences in sting nematode abundance among fluensulfone rates or the 1,3-D treatment (Fig. 1). At harvest in 2016, there were more sting nematodes at the lowest rate of fluensulfone than the 1,3-D treatment. Nematicide treatments decreased sting nematode abundance at harvest by 52 to 91%, 66 to 100%, and 55 to 84% in 2016, 2017, and 2018, respectively.

**Figure 2:**
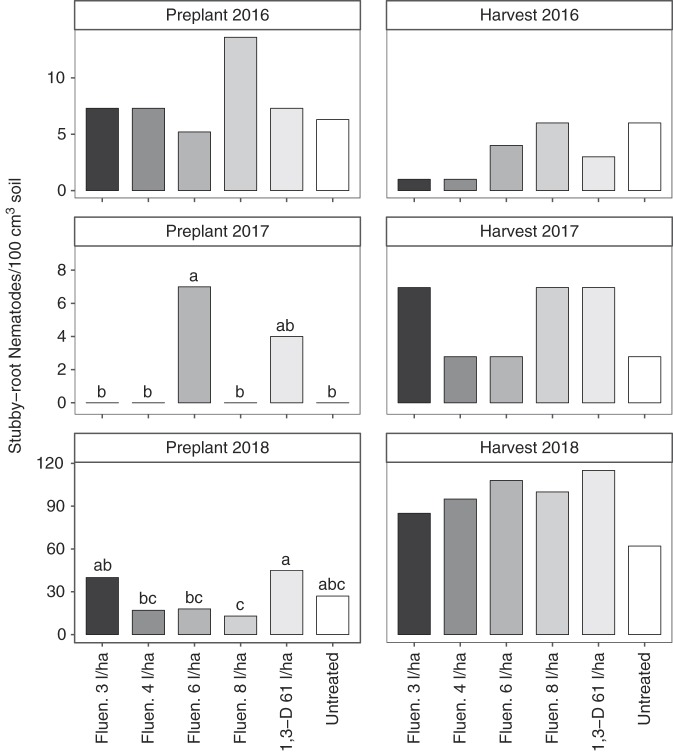
Stubby-root nematode (*Paratrichodorus* sp.) abundance as affected by nematicide treatments. The abbreviations Fluen. and 1,3-D denote fluenosulfone (Nimitz EC) and 1,3-dichloropropene nematicide applications, respectively. Different letters indicate significantly different means based on Fisher’s protected LSD at *α* = 0.05. Bars represent means of six replicates.

Stubby-root nematodes were present at very low abundance in 2016 and 2017 and were not affected by nematicide treatments except before planting in 2017 (Fig. 2). In 2018, stubby-root nematode abundances varied somewhat across treatments before planting. Fewer stubby-root nematodes were present at harvest than before planting, but were not affected by nematicide treatments at harvest.

**Figure 3:**
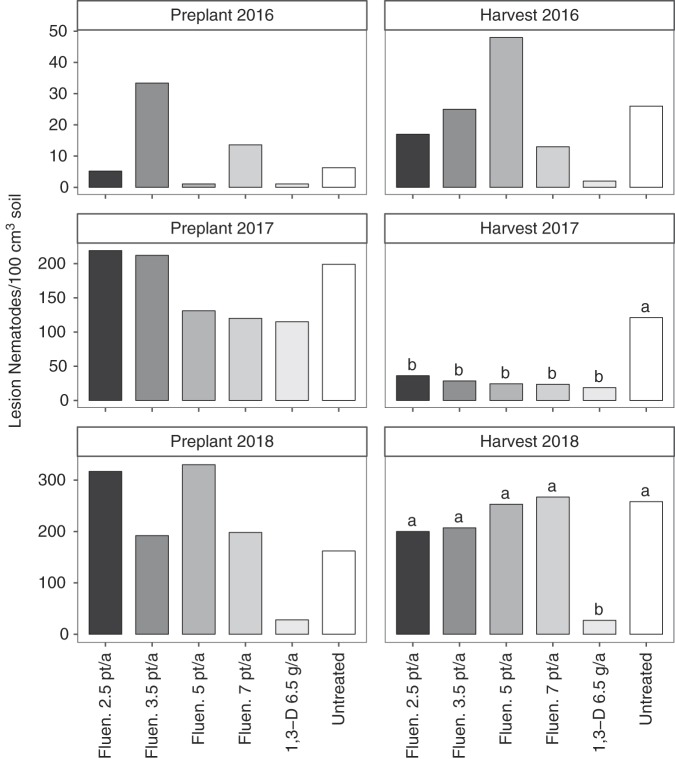
Lesion nematode (*Pratylenchus* sp.) abundance as affected by nematicide treatments. The abbreviations Fluen. and 1,3-D denote fluenosulfone (Nimitz EC) and 1,3-dichloropropene nematicide applications, respectively. Different letters indicate significantly different means based on Fisher’s protected LSD at *α* = 0.05. Bars represent means of six replicates.

Lesion nematode abundances were not significantly different among nematicide treatments before planting in any year (Fig. 3). Nematicide impacts on final lesion nematode abundances varied among years. There were no significant effects in 2016, all nematicides effectively reduced lesion nematodes compared with the untreated control in 2017, and 1,3-D reduced lesion nematode abundances compared with any other treatment in 2018. Stunt nematode were present in large numbers in 2018 (219 and 888 nematodes/100 cm^3^ soil before planting and at harvest, respectively), but their numbers were not significantly affected by nematicide treatments.

**Figure 4:**
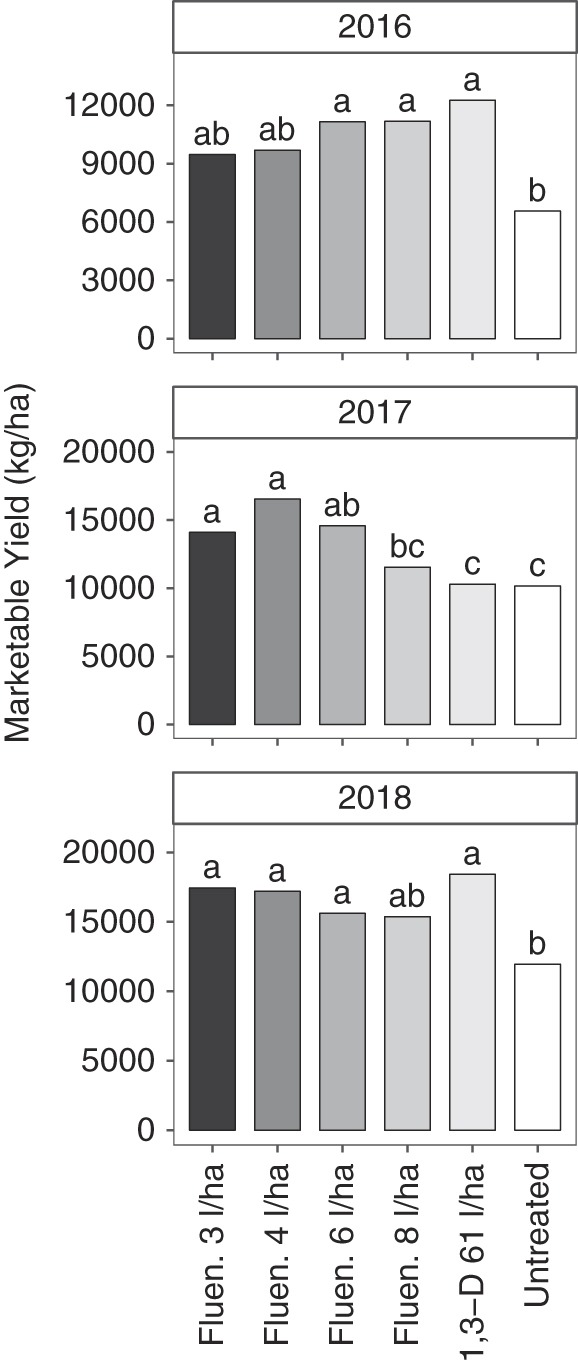
Marketable potato yield (excluding culls) as affected by nematicide treatments in 2016, 2017, and 2018. The abbreviations Fluen. and 1,3-D denote fluensulfone (Nimitz EC) and 1,3-dichloropropene nematicide applications, respectively. Different letters indicate significantly different means based on Fisher’s protected LSD at *α* = 0.05. Bars represent means of six replicates.

Total marketable yield of potato was significantly affected by nematicide treatments each year, but trends varied by year (Fig. 4). In 2016 and 2018, marketable yields were generally greater for fluensulfone or 1,3-D treatments than the untreated control, but did not vary among fluensulfone or 1,3-D treatments. In 2016, lower rates of fluensulfone (3 and 4 l/ha) had statistically similar yields to the untreated control and, in 2018, fluensulfone at 8 l/ha was not different from the untreated control. In 2016, fluensulfone (6 and 8 l/ha) and 1,3-D increased marketable potato yield 5,800 to 6,000 kg/ha or 49 to 66% relative to the untreated control. In 2018, fluensulfone (3, 4, and 6 l/ha) and 1,3-D increased marketable potato yield 4,400 to 7400 kg/ha or 33 to 55%. The trend was different in 2017 as marketable yields were greater at low fluensulfone rates (3, 4, and 6 l/ha) than the untreated control, but fluensulfone at 8 l/ha and 1,3-D treatments had similar yields to control and significantly lesser yield than 3 and 4 l/ha fluensulfone. In 2017, fluensulfone (3, 4, and 6 l/ha) increased marketable potato yields 5,300 to 7,800 kg/ha or 41 to 61% relative to the untreated control.

When potato yield was analyzed by size grade, nematicides affected primarily grade A yield and trends were generally similar to trends for total marketable yield (Table 4). In 2016, nematicides significantly affected A1, A2, and A123 with yields generally significantly lesser for control than 1,3-D and greater fluensulfone rates. This was similar to overall trends for total marketable yield. In 2017, nematicides affected all grade A yield categories. Yields were generally lesser for 1,3-D and greater fluensulfone rates than lesser fluensulfone rates which was similar to overall trends for total marketable yield. In 2018, nematicides affected grades A1 and A123 yields with yields greater for fluensulfone at 3 and 4 l/ha and 1,3-D than the untreated control. Grades A1 and A123 yields for fluensulfone at 6 and 8 l/ha were not significantly different than the untreated control. Nematicides did not significantly affect grade C yields in any year or grade B yields in 2016 or 2017. In 2018, grade B yields were significantly greater for fluensulfone at 3 l/ha than the untreated control, but not different among other treatments.

**Table 4.** Marketable potato tuber yield (kg/ha) by grade as affected by nematicide treatments.^a^

Grade	C^b^	B	A1	A2	A3	A123
Nematicide treatment	2016					
Fluensulfone 3 l/ha	547	1,095	7,174 ab	441 b	220	7,835 ab
Fluensulfone 4 l/ha	547	1,166	6,563 ab	1,223 ab	192	7,977 ab
Fluensulfone 6 l/ha	576	1,180	7,551 a	1,849 a	0	9,399 a
Fluensulfone 8 l/ha	704	1,315	7,985 a	1,130 ab	50	9,165 a
1,3-dichloropropene 61 l/ha	597	1,230	8,418 a	1,969 a	43	10,430 a
Untreated control	661	939	4,721 b	242 b	0	4,963 b
Nematicide treatment	2017					
Fluensulfone 3 l/ha	594	1,299	8,774 ab	2,167 ab	1,275 ab	12,216 ab
Fluensulfone 4 l/ha	679	1,505	9,665 a	2,842 a	1,851 a	14,357 a
Fluensulfone 6 l/ha	771	1,244	9,503 a	1,353 bc	1,706 a	12,562 ab
Fluensulfone 8 l/ha	540	997	7,648 abc	1,079 c	1,285 ab	10,011 bc
1,3-dichloropropene 61 l/ha	712	1,313	6,029 c	1,613 bc	634 bc	8,276 c
Untreated control	492	1,239	6,611 bc	1,359 bc	467 c	8,437 c
Nematicide treatment	2018					
Fluensulfone 3 l/ha	3,235	6,143 a	7,160 a	533	313	8,006 a
Fluensulfone 4 l/ha	3,427	5,567 ab	7,430 a	675	284	8,383 a
Fluensulfone 6 l/ha	3,263	5,610 ab	6,051 ab	633	114	6,797 ab
Fluensulfone 8 l/ha	3,214	5,524 ab	6,079 ab	398	114	6,591 ab
1,3-dichloropropene 61 l/ha	3,647	5,809 ab	8,006 a	626	277	8,909 a
Untreated control	3,512	4,494 b	3,868 b	100	43	4,010 b

Note: ^a^Different letters in the same column indicate significantly different means based on Fisher’s protected LSD at *α* = 0.05; ^b^tuber grades of C, B, A1, A2, and A3 include harvest potatoes with diameters of 1.3 to 3.7, 3.8 to 4.7, 4.8 to 6.3, 6.4 to 8.3, and 8.4 to 10.2 cm respectively. A123 is total yield for grades A1, A2, and A3 combined.

Nematicide application did not significantly affect cull weight in any category in 2016 or 2017 (Table 5). In 2018, cull weight of rotted tubers was significantly greater for 1,3-D than fluensulfone at 4 l/ha or the untreated control and misshapen cull weight was significantly greater for 1,3-D than any treatment except fluensulfone at 6 l/ha. Incidence of internal defects was low (generally less than 1%) and nematicide application did not affect incidence of internal defects consistent with CRS, internal heat necrosis, or hollow heart in any year.

**Table 5.** Cull potato tuber weight (kg/ha) by category as affected by nematicide treatments.^a^

	Total	Rotted	Green	Growth crack	Misshapen
Nematicide treatment	2016				
Fluensulfone 3 l/ha	6,100	6,100	0	0	0
Fluensulfone 4 l/ha	6,157	6,157	0	0	0
Fluensulfone 6 l/ha	6,719	6,719	0	0	0
Fluensulfone 8 l/ha	6,385	6,385	0	0	0
1,3-dichloropropene 61 l/ha	7,266	7,266	0	0	0
Untreated control	5,091	5,091	0	0	0
Nematicide treatment	2017				
Fluensulfone 3 l/ha	4,639	1,779	1,024	930	907
Fluensulfone 4 l/ha	4,001	1,642	1,246	759	352
Fluensulfone 6 l/ha	3,504	1,397	855	444	807
Fluensulfone 8 l/ha	2,945	851	821	675	597
1,3-dichloropropene 61 l/ha	3,271	1,679	542	430	619
Untreated control	2,601	880	787	553	382
Nematicide treatment	2018				
Fluensulfone 3 l/ha	1,522	1,159 ab	64	78	228 c
Fluensulfone 4 l/ha	1,237	761 b	206	107	164 c
Fluensulfone 6 l/ha	2,190	1,379 ab	220	78	519 ab
Fluensulfone 8 l/ha	1,586	946 ab	263	85	313 bc
1,3-dichloropropene 61 l/ha	2,481	1,529 a	185	107	654 a
Untreated control	1,436	825 b	199	36	377 bc

Note: ^a^Different letters in the same column indicate significantly different means based on Fisher’s protected LSD at α = 0.05.

## Discussion

Both 1,3-D and fluensulfone were effective for managing sting nematode. Fluensulfone provided a similar level of sting nematode control to 1,3-D and, in general, there was not a rate response to fluensulfone. Based on these results, growers may be able achieve sting nematode control at reduced rates of fluensulfone application. The efficacy of 1,3-D for sting nematode management in potato production is well-documented (Weingartner and Shumaker, 1990a, 1990b, 1990d; Crow et al., 2000a), but this report of fluensulfone efficacy against sting nematodes in potato production is novel.

Neither fluensulfone nor 1,3-D were any more effective than the untreated control for managing stubby-root nematodes. Stubby-root nematode abundances were low in 2016 to 2017, which could have obscured any treatment effects, and CRS incidence was also low in this study. However, these results suggest that more work is needed to identify products for managing stubby-root nematodes and CRS. In general, stubby-root nematodes seem to be less susceptible to nematicides than other nematodes, such as sting nematode, based on this and previous studies in Florida potato production (Weingartner and Shumaker, 1990a, 1990b, 1990c, 1990d). Management of stubby-root nematodes and CRS has generally been better with non-fumigant nematicides, particularly carbamates such as aldicarb and oxamyl, compared with fumigants (Weingartner and Shumaker, 1990c; Weingartner et al., 1993). The potato label cancellation of aldicarb has left growers with few chemical options for managing CRS, so additional management options are needed.

Lesion nematode control was better with 1,3-D than fluensulfone, but was inconsistent from year to year with both products. Lesion nematode is an important pathogen in the northern USA as it is associated with potato early dying complex in conjunction with the soil fungus *Verticillium* (Belair et al., 2006; LaMondia, 2006; MacGuidwin et al., 2012), but this complex is not known to be present in the Southeast. The impact of lesion nematode on potato yield in the Southeast is relatively unknown, but it has not been tied to yield suppression in this study or previous studies (Crow et al., 2000). Therefore, from a practical perspective, management of sting and stubby-root nematodes is a higher priority than lesion nematode for growers.

The varying results for the three nematode genera in this study suggest that efficacy of fluensulfone and 1,3-D can vary by target genera. Previous fluensulfone efficacy studies have focused primarily on root-knot nematodes, against which it has generally been effective (Oka et al., 2012, 2013; Morris et al., 2015, 2016; Jones et al., 2017; Castillo et al., 2017). There are few reports on fluensulfone efficacy against nematodes other than root-knot nematodes, but in those studies, efficacy has varied more than for root-knot nematodes reinforcing the results of this study that fluensulfone efficacy varies by target nematode (Oka, 2014; Norshie et al., 2016). In one study, fluensulfone had some efficacy against lesion nematodes (*Pratylenchus penetrans* and *Pratylenchus thornei*) in vitro and in greenhouse soil pots, but efficacy was considered poorer against lesion than root-knot nematodes (Oka, 2014). Fluensulfone was not effective against *Bursaphelenchus xylophilus*, *Ditylenchus dipsaci*, *Aphelenchoides fragariae*, and *Aphelenchoides besseyi* (Oka, 2014). Against potato cyst nematode (*Globodera pallida*) in field trials in England, fluensulfone was effective as a granular formulation and as an emulsifiable concentrate, and efficacy was comparable to or worse than oxamyl and fosthiazate, standard products in that region (Norshie et al., 2016).

Both 1,3-D and fluensulfone were effective for improving potato productivity except in 2017 when 1,3-D and high rates of fluensulfone (8 l/ha) produced yields similar to the untreated control and less than low fluensulfone rates. Impaired plant growth from pesticide application (phytotoxicity) is an explanation for lower yield in that case, although no visual symptoms of potato phytotoxicity were observed in 2017. Both fluensulfone and 1,3-D can be phytotoxic at certain rates to some crops (Desaeger and Csinos, 2005; Morris et al., 2016). Longer time periods between nematicide application and planting allow pesticides to dissipate, reducing the likelihood of phytotoxicity. The shorter time period between nematicide application and planting in 2017 (15 d) relative to 2016 and 2018 (21 and 27 d, respectively) could be a contributing factor to the potential phytotoxicity observed in 2017, but further research is needed to validate this. Excess soil moisture and low temperatures also increase risk of phytotoxicity by increasing nematicide persistence, particularly fumigants such as 1,3-D. However, that does not appear to be a substantial factor in this study since among years this study was conducted, conditions around nematicide application were driest and warmest in 2017, when potential phytotoxicity occurred.

In general, fluensulfone performed similarly to 1,3-D for improving potato productivity in this study. There was not a significant positive fluensulfone rate response for potato productivity or nematode management. This suggests the lower fluensulfone rates tested in this study may be adequate for sting nematode management, which would be economically advantageous for growers. Rate response has been inconsistent in previous fluensulfone studies. In a field study involving potato cyst nematode, fluensulfone was more effective at greater rates when applied as a granular formulation (Norshie et al., 2016). In some in vitro trials, fluensulfone efficacy against *Meloidogyne javanica* increased as fluensulfone rate increased (Oka et al., 2013). Foliar applications of fluensulfone were more effective for managing root-knot nematodes at greater rates in two separate vegetable greenhouse studies (Oka et al., 2012; Morris et al., 2016). In Florida tomato (*Solanum lycopersicum*) production, two rates of fluensulfone (1.96 and 2.80 kg a.i./ha) applied through drip irrigation performed similarly (Castillo et al., 2017). In greenhouse experiments with lima bean (*Phaseolus lunatus*), two rates of fluensulfone (1.64 and 2.34 l a.i./ha) were similarly effective for managing *Meloidogyne incognita* (Jones et al., 2017).

In summary, fluensulfone was an effective non-fumigant nematicide for sting nematode management in potato production. Results of this study suggest that it may perform at a similar level to 1,3-D for sting nematode management. Fluensulfone was generally equally effective among rates tested, suggesting nematode management may be achieved at lower rates, which is economically advantageous for growers. Fluensulfone was not as effective against lesion or stubby-root nematodes, suggesting that efficacy varies by target nematode. Continued research is needed to evaluate and expand options for controlling stubby-root nematodes as they are an important vector of CRS. Yield suppression by high rates of fluensulfone (8 l/ha) and 1,3-D, albeit only in one of three years, and potential phytotoxicity warrants monitoring.
